# The Glucagon-Like Peptide-1 Receptor Agonist Exendin-4 Inhibits Lipopolysaccharide-Induced Osteoclast Formation and Bone Resorption via Inhibition of TNF-*α* Expression in Macrophages

**DOI:** 10.1155/2018/5783639

**Published:** 2018-03-13

**Authors:** Wei-Ren Shen, Keisuke Kimura, Masahiko Ishida, Haruki Sugisawa, Akiko Kishikawa, Kazuhiro Shima, Saika Ogawa, Jiawei Qi, Hideki Kitaura

**Affiliations:** Division of Orthodontics and Dentofacial Orthopedics, Department of Translational Medicine, Tohoku University Graduate School of Dentistry, 4-1 Seiryo-machi, Aoba-ku, Sendai 980-8575, Japan

## Abstract

Glucagon-like peptide-1 (GLP-1) receptor agonists are an effective treatment approach for type 2 diabetes. Recently, anti-inflammatory effects of GLP-1 receptor agonists have also been reported. Lipopolysaccharide (LPS) induces inflammation and osteoclast formation. In this study, we investigated the effect of exendin-4, a widely used GLP-1 receptor agonist, in LPS-induced osteoclast formation and bone resorption. LPS with or without exendin-4 was administered on mouse calvariae by daily subcutaneous injection. The number of osteoclasts, the ratio of bone resorption pits, and the level of C-terminal cross-linked telopeptide of type I collagen (CTX) were significantly lower in LPS- and exendin-4-coadministered mice than in mice administered with LPS alone. RANKL and TNF-*α* mRNA expression levels were lower in the exendin-4- and LPS-coadministered group than in the LPS-administered group. Our *in vitro* results showed no direct effects of exendin-4 on RANKL-induced osteoclast formation, TNF-*α*-induced osteoclast formation, or LPS-induced RANKL expression in stromal cells. Conversely, TNF-*α* mRNA expression was inhibited in the exendin-4- and LPS-cotreated macrophages compared with cells treated with LPS alone. These results indicate that the GLP-1 receptor agonist exendin-4 may inhibit LPS-induced osteoclast formation and bone resorption by inhibiting LPS-induced TNF-*α* production in macrophages.

## 1. Introduction

The prevalence of type 2 diabetes mellitus is increasing worldwide, and the condition has become a major public health problem. Individuals with type 2 diabetes have been shown to have a higher risk of bone fracture compared with individuals without type 2 diabetes [1]. This higher risk might be associated with the pathobiology of type 2 diabetes itself; however, the underlying mechanisms remain unclear [2]. Additionally, increased bone fracture risk is a consequence of therapeutic regimen used to treat hyperglycemia [3]. For example, patients treated with thiazolidinediones and human recombinant insulin have been shown to have an increased bone fracture risk [4–7]. Conversely, treatment with metformin is related to decreased bone fracture risk [8].

Osteoclast recruitment is crucial to the pathogenesis of diseases involving bone erosion, such as rheumatoid arthritis [9]. Osteoclasts derived from bone marrow cells are responsible for bone resorption and remodeling [10]. Receptor activator of NF-kB ligand (RANKL) and macrophage colony-stimulating factor (M-CSF) are two key factors required for osteoclast formation and activation [11]. Independent of RANKL, tumor necrosis factor- (TNF-) *α* has also been reported to induce osteoclast formation *in vitro* [12–14] and *in vivo* [15, 16].

Lipopolysaccharide (LPS) strongly induces inflammation and inflammatory bone loss [17–21]. LPS has also been found to induce production of proinflammatory cytokines, such as TNF-*α*, from macrophages or other cells at the site of inflammation [22, 23]. Such proinflammatory cytokines have been reported to be involved in LPS-induced osteoclast formation and bone destruction in *in vivo* and *in vitro* studies [18, 24–27]. Additionally, LPS can stimulate osteoblasts to produce or secrete RANKL [28].

Glucagon-like peptide-1 (GLP-1), an intestinal hormone, plays important roles in blood glucose control and proliferation of pancreatic islet *β*-cells [29, 30]. GLP-1 receptor-deficient mice were reported to exhibit osteopenia and increased osteoclast formation, suggesting that the GLP-1 signaling has an inhibitory effect of bone resorption on bone metabolism [31]. An anabolic effect of GLP-1 on bone metabolism has also been proposed. GLP-1 receptor activation has been shown to induce bone formation in streptozotocin-induced diabetic and fructose-stimulated insulin-resistant rats [32].

It has been reported that patients with type 2 diabetes have high risk of bone fracture [1, 2]. Furthermore, antidiabetic medicines such as thiazolidinediones may further promote bone resorption and increase fracture risk [33–35]. However, a recent meta-analysis has reported that GLP-1 receptor agonist treatment does not affect fracture risk in type 2 diabetic patients [36, 37].

The anabolic and antiresorptive effects of GLP-1 receptor suggest that GLP-1 receptor signaling may be a promising therapeutic target for osteoporosis or other osteolytic bone diseases; such a therapeutic approach would be facilitated by the fact that the first commercially available GLP-1 receptor agonist, exendin-4, has already been approved for the treatment of diabetes for over 10 years [38]. Exendin-4 shares similar structural and functional properties to GLP-1 but is resistant to the degradation by dipeptidyl peptidase-IV, which can degrade GLP-1 immediately in the blood [39]. The extended half-life, improved pharmacokinetics, and high potency of exendin-4 make it suitable for clinical use [39–41].

In the present study, we investigated the effects of exendin-4 on LPS-induced osteoclast formation and bone remodeling in mice.

## 2. Materials and Methods

### 2.1. Animals and Reagents

Eight- to ten-week-old male C57BL6/J mice were obtained from CLEA Japan (Tokyo, Japan) and maintained at our animal facility. All animal care and experiments were conducted according to Tohoku University rules and regulations. Four mice were randomly assigned to each experimental group. Both *Escherichia coli* LPS and exendin-4 were purchased from Sigma-Aldrich (St. Louis, MO).

### 2.2. Histological Analysis

A previous *in vivo* study demonstrated that daily subcutaneous injections of 100 *μ*g LPS to mouse calvariae for 5 days effectively induced osteoclast formation [42]. Therefore, we followed the same protocol, dose, and LPS administration period in this study. The mice were divided into four experimental groups and subjected to daily subcutaneous injections on the calvaria with phosphate-buffered saline (PBS, negative control group), LPS alone (100 *μ*g/day, positive control group), LPS (100 *μ*g/day) and exendin-4 (20 *μ*g/day), and exendin-4 alone (20 *μ*g/day) for 5 days. All mice calvariae were excised immediately after sacrifice on the sixth day. The calvariae were fixed in 4% PBS-buffered formaldehyde at 4°C overnight and then demineralized with 14% ethylenediaminetetraacetic acid (EDTA) at room temperature for three days. Each calvaria was cut into three pieces perpendicular to the sagittal suture. Samples were then embedded in paraffin and cut into 5 *μ*m sections using a microtome. The paraffin sections were stained with tartrate-resistant acid phosphatase (TRAP) solution prepared by mixing acetate buffer (pH 5.0), naphthol AS-MX phosphate (Sigma Chemical, St. Louis, MO, USA), Fast Red Violet LB Salt (Sigma), and 50 mM sodium tartrate. The sections were counterstained with hematoxylin. Osteoclasts were defined in this study as TRAP-positive cells with three or more nuclei. We counted the number of osteoclasts only at the suture mesenchyme of the sagittal suture in all slides according to the method in our previous work [43].

### 2.3. Preparation of Osteoclast Precursors for Osteoclastogenesis

To isolate bone marrow cells from C57BL6/J mice, femora and tibiae were aseptically removed after sacrifice. The epiphyses of these long bones were removed, and the bone marrow was flushed into a sterile Petri dish with a 25-gauge needle and 10 ml syringe filled with culture medium. The bone marrow was then filtered with a 40 *μ*m nylon cell strainer (Falcon, USA) and centrifuged. The harvested cells were incubated in a culture medium comprising alpha-modified minimal essential medium (*α*-MEM; Sigma) containing 10% fetal bovine serum (FBS), 100 IU/ml penicillin G (Meiji Seika, Tokyo, Japan), and 100 *μ*g/ml streptomycin (Meiji Seika), with M-CSF added. Nonadherent cells were removed by washing with PBS, and adherent cells were harvested using trypsin-EDTA solution (Sigma-Aldrich). The harvested cells were seeded and further cultured in the presence of M-CSF. Adherent cells were used as osteoclast precursors in this study as previously reported [43]. Osteoclast precursors were seeded at 5 × 10^4^ cells per 200 *μ*l of medium in a 96-well plate and cultured in medium containing M-CSF alone (100 ng/ml), M-CSF (100 ng/ml) and RANKL (100 ng/ml) or TNF-*α* (100 ng/ml), M-CSF (100 ng/ml) and RANKL (100 ng/ml) or TNF-*α* (100 ng/ml) with exendin-4 (100 ng/ml), and M-CSF (100 ng/ml) with exendin-4 (100 ng/ml), for 5 days. The cultured cells were then fixed with 10% formalin for 30 min. After fixation, the cells were permeabilized with 0.2% Triton X-100 for 5 min at room temperature, then incubated in TRAP staining solution prepared as described above. TRAP-positive cells with three or more nuclei were considered to be osteoclasts and were counted under a light microscope.

### 2.4. Preparation of Bone Marrow Stromal Cells

Bone marrow cells were obtained by the method described above and cultured in Dulbecco's modified Eagle's medium (DMEM; Sigma) containing 10% FBS, 100 IU/ml penicillin G (Life Technologies, Carlsbad, CA), and 100 *μ*g/ml streptomycin (Life Technologies) for two weeks. Then the culture disks were washed vigorously with PBS to remove nonadherent cells. Adherent cells were used as stromal cells in this study as previously reported [43].

### 2.5. Isolation of Murine Macrophages

Macrophages were obtained from the peritoneal cavity of mice. To obtain resident macrophages under resting conditions, we injected 5 ml of sterile ice-cold PBS (pH 7.4) into the peritoneal cavity and aspirated the fluid to harvest peritoneal cells. The cells were washed twice with *α*-MEM medium (Sigma) containing 10% FBS. After 1 hour of culture, nonadherent cells were removed, and after 24 hours of culture, adherent cells were harvested and used as macrophages.

### 2.6. Isolation of RNA and Real-Time RT-PCR Analysis

Calvariae from the *in vivo* experiments were frozen in liquid nitrogen and crushed by Micro Smash MS-100R (Tomy Seiko, Tokyo, Japan) in 800 *μ*l TRIzol reagent (Invitrogen, Carlsbad, CA) for each sample. Total RNA was extracted with an RNeasy mini kit (Qiagen, Valencia, CA) according to the manufacturer's protocol. For the *in vitro* experiments, bone marrow stromal cells or macrophages were incubated in culture medium supplemented with PBS, LPS (100 ng/ml), LPS (100 ng/ml) and exendin-4 (100 ng/ml), and exendin-4 (100 ng/ml). After three days of culture, total RNA was isolated from adherent cells. Total RNA of stromal cells or peritoneal macrophages was isolated using an RNeasy mini kit (Qiagen). cDNA was synthesized for each sample from 2 *μ*g total RNA with oligo-dT primers (Invitrogen) and reverse transcriptase in a total volume of 20 *μ*l. The corresponding expression levels of RANKL and TNF-*α* mRNA were evaluated by real-time RT-PCR using a Thermal Cycler Dice Real Time System (Takara, Shiga, Japan). Each reaction comprised a total volume of 25 *μ*l containing 2 *μ*l cDNA and 23 *μ*l of a mixture of SYBR Premix Ex Taq (Takara) and 50 pmol/*μ*l primers. The PCR cycling conditions were as follows: 95°C for 10 s for initial denaturation followed by 45–60 amplification cycles, with each cycle comprising a denaturation step of 95°C for 5 s and then an annealing step of 60°C for 30 s. Relative expression levels of TNF-*α* and RANKL mRNAs were calculated by normalization to glyceraldehyde 3-phosphate dehydrogenase (GAPDH) mRNA levels. The primer sequences used for cDNA amplification were as follows: 5′-GGTGGAGCCAAAAGGGTCA-3′ and 5′-GGGGGCTAAGCAGTTGGT-3′ for GAPDH; 5′-AGGCGGTGCTTGTTCCTCA-3′ and 5′-AGGCGAGAAGATGATCTGACTGCC-3′ for TNF-*α*; and 5′-CCTGAGGCCAGCCATTT-3′ and 5′-CTTGGCCCAGCCTCGAT-3′ for RANKL as already reported [43].

### 2.7. Micro-CT Imaging and Analysis for Bone Destruction Area

We obtained mouse calvariae immediately after sacrifice. The calvariae were fixed in 4% PBS-buffered formaldehyde at 4°C for 3 days. To assess the bone resorption pits on the calvariae, samples were washed thoroughly with PBS and scanned with microfocus computed tomography (ScanXmate-E090, Comscan, Kanagawa, Japan). TRI/3D-BON64 software (RATOC System Engineering, Tokyo, Japan) was used to create three-dimensional images of the mouse calvariae, and the ratio of bone resorption area to total area was measured by ImageJ (NIH, Bethesda, MD) as previously reported [43].

### 2.8. Measurement of Serum CTX (C-Terminal Cross-Linked Telopeptide of Type I Collagen) Value

Blood was collected with microhematocrit tubes from the orbital sinuses of the mice after 5 days of daily administration of PBS, LPS with or without exendin-4, or exendin-4 alone. The levels of CTX were determined using a mouse C-terminal telopeptide of type I collagen assay kit (IDS, Tyne and Wear, UK). Levels of C-terminal telopeptide of type I collagen were assessed by measuring absorbance at 450 nm with a microplate reader (Remote Sunrise; Tecan, Japan), with 620 nm as the reference wavelength.

### 2.9. Cell Viability Assay for Osteoclast Precursors

Osteoclast precursors were seeded in a 96-well plate (1 × 10^4^ cells in 200 *μ*l medium per well) and incubated with M-CSF (100 ng/ml) with or without exendin-4 (100 ng/ml). After 5 days of incubation, the cells were washed with PBS and cultured in 100 *μ*l culture medium of each well. Four replicates were assessed for each sample. Then, 10 *μ*l cell counting kit-8 (Dojin, Kumamoto, Japan) solution was added to each well, and the plate was further incubated for 2 h at 37°C. Absorbance at 450 nm was measured by a microplate reader for each well as previously reported [43].

### 2.10. Statistical Analysis

Data are expressed as means ± standard deviation. The statistical significance of differences between groups was determined by Scheffe's test. *P* < 0.05 was considered significant.

## 3. Results

### 3.1. *In Vivo* Inhibitory Effect of Exendin-4 on LPS-Induced Osteoclast Formation

**Figure 1:**
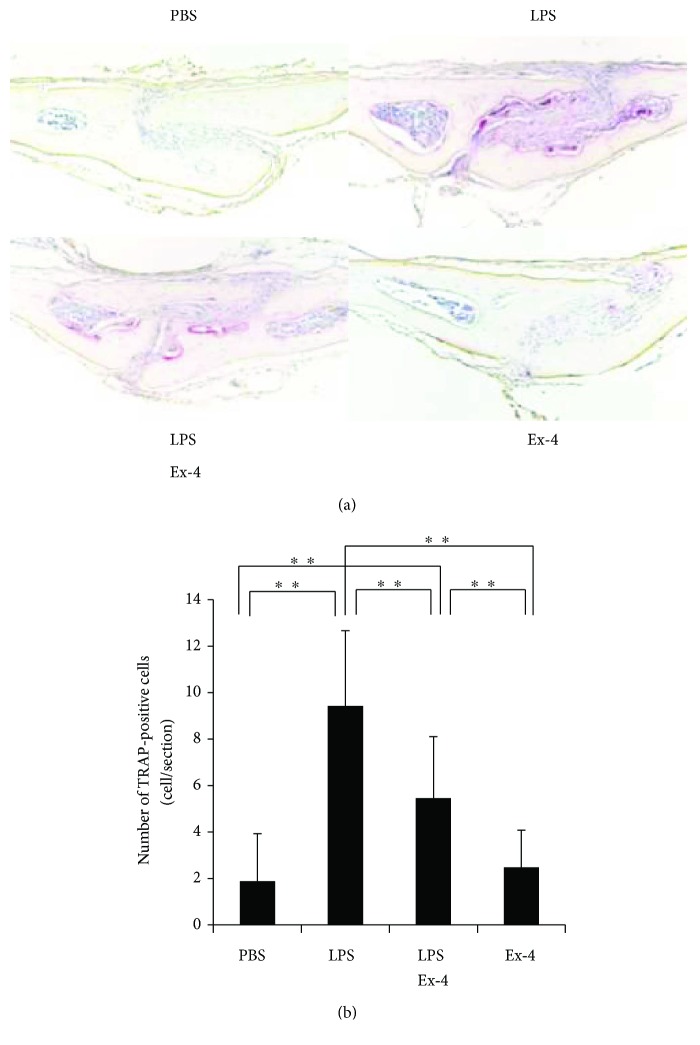
*In vivo* effect of exendin-4 on lipopolysaccharide- (LPS-) induced osteoclast formation. (a) Histological sections of mouse calvariae after 5-day daily supracalvarial injections with phosphate-buffered saline (PBS), LPS (100 *μ*g/day), LPS (100 *μ*g/day) with exendin-4 (20 *μ*g/day), and exendin-4 (20 *μ*g/day). Tartrate-resistant acid phosphatase (TRAP) staining and hematoxylin counterstaining were performed. TRAP-positive cells were stained dark red. (b) The numbers of TRAP-positive cells in the suture mesenchyme of calvaria from the mouse groups administered with PBS, LPS, LPS with exendin-4, and exendin-4, respectively. Data is expressed as means ± standard deviation (SD). Statistical significance were determined by Scheffe's test (*n* = 4; ^∗∗^*p* < 0.01).

We injected LPS with or without exendin-4 on mouse calvariae to analyze the effect of exendin-4 on LPS-induced osteoclast formation *in vivo*. After LPS administration for 5 consecutive days, many large multinucleated osteoclasts were observed within the suture mesenchyme in the histological sections. However, the mean number of osteoclasts was significantly lower in the LPS- and exendin-4-coadministered group than in the group administered with LPS alone (Figures 1(a) and 1(b)).

### 3.2. *In Vivo* Inhibitory Effect of Exendin-4 on LPS-Induced Bone Resorption

**Figure 2:**
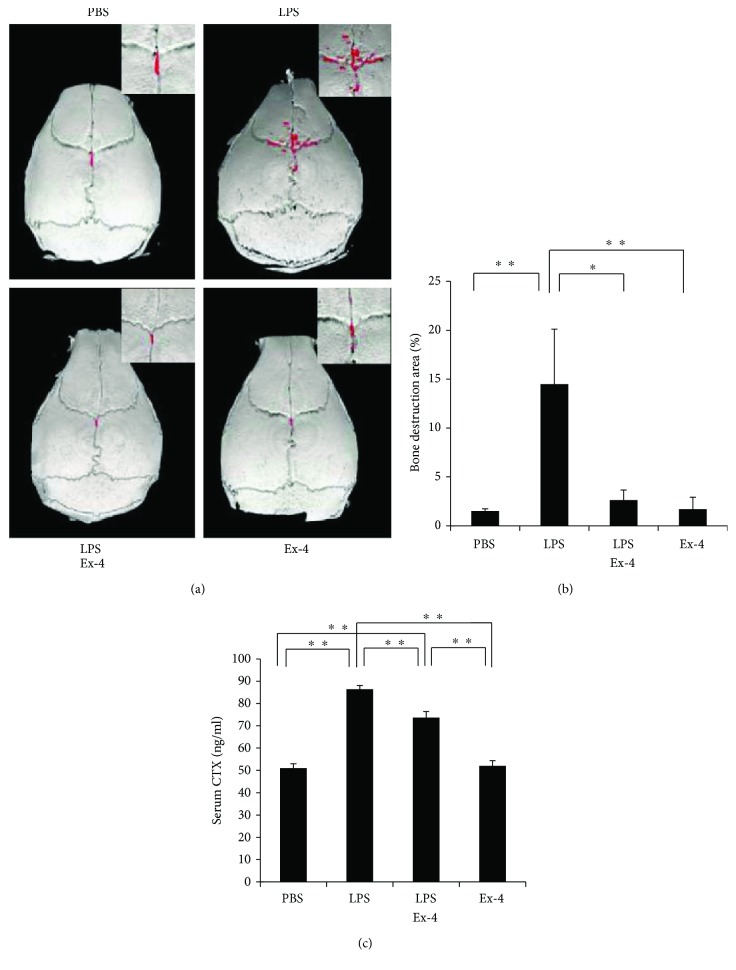
Exendin-4 inhibited LPS-induced bone resorption *in vivo*. (a) 3D reconstructed images of calvariae from micro-CT scanning. Mice were subjected to 5-day daily subcutaneous injections on the calvariae with PBS, LPS (100 *μ*g/day) with or without exendin-4 (20 *μ*g/day), and exendin-4 (20 *μ*g/day), and calvariae were excised on the sixth day. The red dots indicate areas of bony destruction. (b) Ratio of bone destruction area to total bone area. Data is expressed as means ± SD (*n* = 4; ^∗^*p* < 0.05, ^∗∗^*p* < 0.01). The statistical significance of differences was determined by Scheffe's test. (c) Serum levels of C-terminal telopeptide of type I collagen (CTX) determined by a mouse CTX assay kit. Data is expressed as means ± SD. The statistical significance of differences was determined using Scheffe's test (*n* = 4; ^∗∗^*p* < 0.01).

The mouse calvariae were scanned with microfocus computed tomography, and the amount of bone resorption areas was compared between each group. Many bone destruction defects were noted in the LPS group. The ratio of the bone resorption area to the total area was significantly higher in the LPS-administered group than in the PBS-administered and exendin-4-administered groups. Moreover, the LPS- and exendin-4-coadministered groups demonstrated less bone destruction than the group administered with LPS alone (Figures 2(a) and 2(b)). Serum levels of C-terminal telopeptide of type I collagen (CTX), a marker of bone resorption, in mouse serum samples were analyzed by a mouse CTX assay kit. The serum CTX level in the LPS-alone-administered group was higher than PBS-administered group. However, the serum CTX level in the LPS- and exendin-4-coadministered group was lower than that in the LPS-alone-administered group (Figure 2(c)).

### 3.3. *In Vivo* Inhibitory Effect of Exendin-4 on the Expression of LPS-Induced Osteoclast-Related Cytokines (TNF-*α* and RANKL)

**Figure 3:**
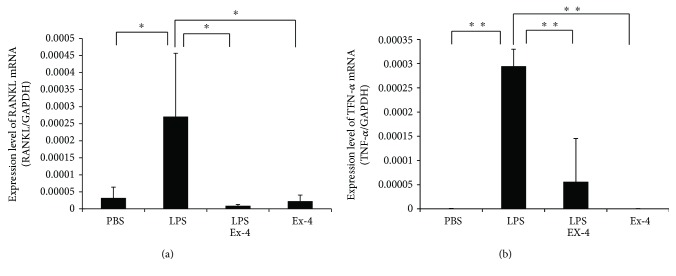
Exendin-4 suppressed expression of LPS-induced tumor necrosis factor- (TNF-) *α* and receptor activator of NF-kB ligand (RANKL) *in vivo*. TNF-*α* and RANKL mRNA levels in mouse calvariae were determined using real-time RT-PCR. Total RNA was isolated from mouse calvariae after 5-day daily supracalvarial injections with PBS, LPS (100 *μ*g/day) with or without exendin-4 (20 *μ*g/day), and exendin-4 alone (20 *μ*g/day). TNF-*α* and RANKL mRNA levels were normalized to the expression of glyceraldehyde 3-phosphate dehydrogenase (GAPDH). Data is expressed as means ± SD. The statistical significance of differences was determined using Scheffe's test (*n* = 4; ^∗^*p* < 0.05, ^∗∗^*p* < 0.01).

Bone chips from mouse calvariae were analyzed by real-time RT-PCR to measure expression levels of TNF-*α* and RANKL mRNA. TNF-*α* and RANKL mRNA levels were elevated in the LPS-administered group compared with the PBS-administered group. Conversely, TNF-*α* and RANKL mRNA expression levels were reduced in the exendin-4- and LPS-coadministered group compared with the LPS-administered group (Figure 3).

### 3.4. Exendin-4 Cannot Affect RANKL-Induced Osteoclast Formation, TNF-*α*-Induced Osteoclast Formation, Cell Viability of Osteoclast Precursor Cells, and LPS-Induced RANKL Expression in Stromal Cells

**Figure 4:**
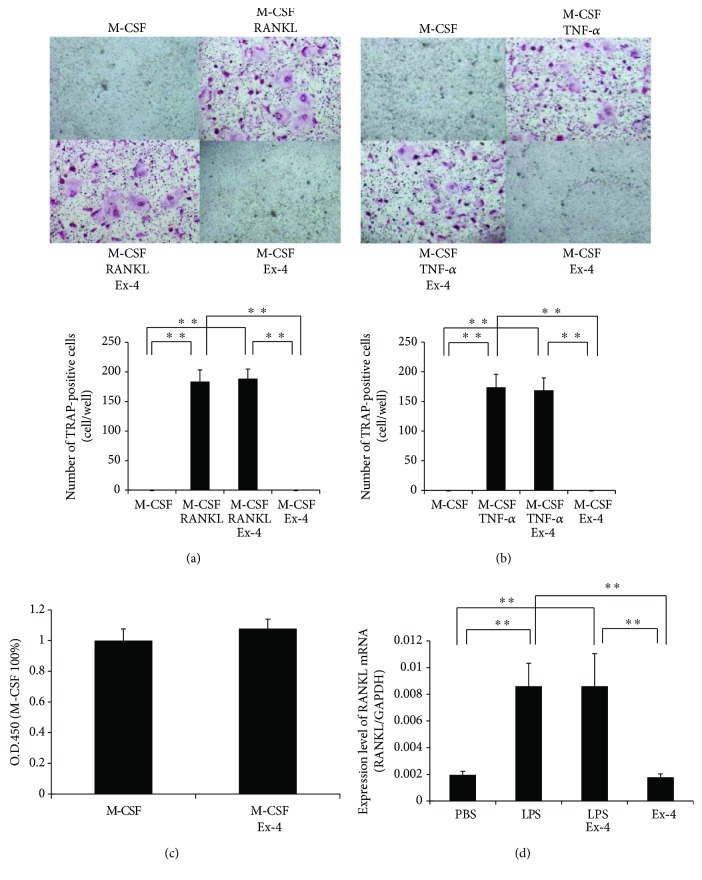
Exendin-4 had no effect on RANKL-induced osteoclast formation, TNF-*α*-induced osteoclast formation, osteoclast precursor cell viability, or LPS-induced RANKL expression in stromal cells *in vitro*. (a) Microscopic images and numbers of TRAP-positive cells. Osteoclast precursors were treated with macrophage colony-stimulating factor (M-CSF) alone, M-CSF with RANKL, M-CSF with RANKL and exendin-4, and M-CSF with exendin-4 for 5 days, then stained with TRAP solution. (b) Microscopic images and numbers of TRAP-positive cells. Osteoclast precursors were treated with M-CSF alone, M-CSF with TNF-*α*, M-CSF with TNF-*α* and exendin-4, and M-CSF with exendin-4 for 5 days, then stained with TRAP solution. (c) Cell viability of osteoclast precursor cells treated with M-CSF alone and M-CSF with exendin-4 for 5 days. Cell viability was determined by cell counting kit-8. Data is presented as percentage activity relative to the activity in the culture with M-CSF alone and is expressed as means ± SD. (d) RANKL mRNA expression levels in stromal cells determined by real-time RT-PCR method. Total RNA was extracted from stromal cells that were cultured with PBS, LPS with or without exendin-4, and exendin-4 alone, respectively. RANKL mRNA levels were normalized to that of GAPDH. Statistical significance of differences was determined by Scheffe's test (*n* = 4; ^∗∗^*P* < 0.01).

To investigate whether exendin-4 affects osteoclast precursor cells directly, we analyzed the effects of exendin-4 on RANKL-induced osteoclast formation, TNF-*α*-induced osteoclast formation, and viability of osteoclast precursors. There were large numbers of TRAP-positive cells among osteoclast precursor cells cultured with M-CSF and RANKL or TNF-*α*. Likewise, TRAP-positive cells were also observed among the osteoclast precursor cells cultured with M-CSF and RANKL or TNF-*α* in the presence of exendin-4 (Figures 4(a) and 4(b)). Additionally, there was no evident difference in cell viability between the two cultures after 5 days of culture (Figure 4(c)). These results indicate that the inhibitory effect of exendin-4 may not be related to a direct action of exendin-4 on the proliferation and differentiation of osteoclast precursors.

We next evaluated whether exendin-4 inhibited LPS-induced RANKL expression in stromal cells *in vitro*. RANKL mRNA expression levels were higher in LPS-treated stromal cells than in control and exendin-4-treated stromal cells. However, stromal cells treated with both LPS and exendin-4 demonstrated similar RANKL mRNA expression levels to those treated with LPS alone (Figure 4(d)). These results show that the inhibitory effect of exendin-4 may not be related to a direct action of exendin-4 on RANKL expression in stromal cells.

### 3.5. Exendin-4 Suppresses LPS-Induced TNF-*α* Expression in Macrophages

**Figure 5:**
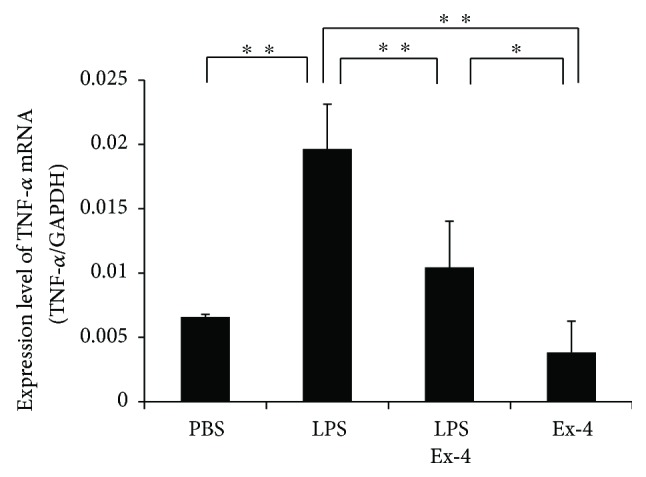
Exendin-4 inhibited LPS-induced expression of TNF-*α* in macrophages. TNF-*α* mRNA levels in macrophages were detected by real-time RT-PCR. Total RNA was isolated from macrophages cultured with PBS, LPS with or without exendin-4, and exendin-4 alone. TNF-*α* mRNA levels were normalized to the levels of GAPDH. Statistical significance of differences was determined by Scheffe's test (*n* = 4; ^∗^*p* < 0.05, ^∗∗^*P* < 0.01).

Real-time RT-PCR was performed to analyze TNF-*α* mRNA expression levels. TNF-*α* mRNA expression was elevated in macrophages treated with LPS alone compared with those treated with PBS. Conversely, TNF-*α* mRNA expression was inhibited in the exendin-4- and LPS-treated macrophages, compared with those treated with LPS alone (Figure 5).

## 4. Discussion

In the present study, we evaluated the effect of the GLP-1 receptor agonist exendin-4 on LPS-induced osteoclast formation and bone-resorption *in vivo*. We found that the GLP-1 receptor agonist inhibited LPS-induced osteoclast formation and bone resorption and also suppressed LPS-induced RANKL and TNF-*α* expression *in vivo*. Conversely, the GLP-1 receptor agonist did not directly inhibit RANKL-induced osteoclast formation, TNF-*α*-induced osteoclast formation, osteoclast precursor cell viability, or LPS-induced RANKL expression in stromal cells *in vitro*. However, the GLP-1 receptor agonist inhibited LPS-induced TNF-*α* expression in macrophages *in vitro*.

GLP-1 plays a crucial role in blood glucose control. To simulate the effect of GLP-1, many GLP-1 analogues and GLP-1 receptor agonists have been developed. The amino acid sequence of the GLP-1 receptor agonist exendin-4 is a modified version of the sequence of GLP-1. Exendin-4 is resistant to degradation by dipeptidyl peptidase-IV and has a much longer plasma half-life than GLP-1 [40], which has a half-life of less than two minutes [39, 41]. The extended half-life, improved pharmacokinetics, and high potency of exendin-4 make it suitable for clinical use [39, 40].

GLP-1 receptor-deficient mice have been reported to exhibit increased bone breakdown, which indicates that GLP-1 receptor signaling is essential to inhibition of osteoclast formation and bone resorption [31]. In the present study, exendin-4 inhibited LPS-induced osteoclast formation. Daily injections of 20 *μ*g of exendin-4 for 5 days (a total of 100 *μ*g) were sufficient to inhibit LPS-induced osteoclast formation *in vivo*. We also evaluated the inhibitory effect of exendin-4 on LPS-induced bone resorption. The extent of bone destruction was determined by the ratio of the destroyed bone area to total bone area, assessed by microfocus computed tomography imaging, and by the serum CTX value of each experimental group. We found that the extent of bone destruction was significantly lower in the LPS- and exendin-4-coadministered group than the group administered with LPS alone. Our results suggest that exendin-4 inhibited LPS-induced osteoclast formation and bone resorption *in vivo*.

In this study, we administered 20 *μ*g/day exendin-4 for 5 days, injected into the supracalvaria. Although previous rodent studies used 20 *μ*g/kg exendin-4 daily for 4 weeks [41, 44], we opted to use a higher dose to enhance the inhibitory effects of exendin-4. Further investigation using clinically relevant doses is needed.

Our findings prompted us to explore the mechanisms contributing to the inhibition of LPS-induced osteoclast formation and bone resorption. We considered two possible mechanisms. First, we considered whether exendin-4 inhibited LPS-induced expression of inflammatory cytokines related to osteoclast formation, such as TNF-*α* and RANKL. Many studies have indicated that LPS induces TNF-*α* and RANKL *in vivo* [28, 45]. RANKL is an essential cytokine for osteoclast formation [10], and it has been reported that TNF-*α* also can induce osteoclast formation *in vivo* [15, 16]. Therefore, it is reasonable to suspect that if levels of both of these cytokines are decreased, osteoclast formation will be inhibited. In the present study, TNF-*α* and RANKL mRNA levels were elevated in the LPS-administered mice. However, this LPS-induced increase in TNF-*α* and RANKL mRNA levels was inhibited in the exendin-4- and LPS-coadministered group, compared with the group administered LPS only. This suggests that one of the mechanisms underlying the inhibitory effect of exendin-4 on LPS-induced osteoclast formation is the inhibition of LPS-induced osteoclast-related cytokines. The other mechanism that we considered was that exendin-4 directly inhibited RANKL- and TNF-*α*-induced osteoclast formation. In the present study, we investigated whether exendin-4 exerted its inhibitory effect on osteoclasts by directly acting on osteoclast precursors. However, exendin-4 did not inhibit RANKL- or TNF-*α*-induced differentiation of osteoclast precursor cells into osteoclasts. Moreover, we investigated whether exendin-4 inhibited osteoclast precursor cell viability. We observed no difference in cell viability between the two groups after 5 days of culture. These results suggest that the inhibitory effect of exendin-4 on osteoclast formation is not due to a direct action of exendin-4 on osteoclast precursors. We then evaluated whether exendin-4 inhibited LPS-induced RANKL expression in stromal cells. Exendin-4 also failed to inhibit LPS-induced RANKL expression in stromal cells. This indicates that inhibition of RANKL expression by exendin-4 may not be due to a direct action of exendin-4 on stromal cells. Finally, we evaluated whether exendin-4 inhibited LPS-induced TNF-*α* expression in macrophages. In our study, exendin-4 inhibited LPS-induced TNF-*α* expression of macrophages. Because TNF-*α* induces osteoclast formation and promotes RANKL expression in stromal cells, our results suggest that the *in vivo* inhibition of LPS-induced osteoclast formation by exendin-4 may be the result of inhibition of LPS-induced TNF-*α* expression in macrophages and subsequent suppression of RANKL expression in stromal cells.

## 5. Conclusions

In conclusion, our results suggested that exendin-4 can inhibit LPS-induced osteoclast formation and bone resorption in vivo. The underlying mechanism may be related to its inhibition in the production of LPS-induced TNF-*α* in macrophages but not related to its direct effect on osteoclast precursors or RANKL expression in stromal cells.
